# Examining Mental Health Knowledge, Stigma, and Service Use Intentions Among Public Safety Personnel

**DOI:** 10.3389/fpsyg.2020.00949

**Published:** 2020-05-29

**Authors:** Rachel L. Krakauer, Andrea M. Stelnicki, R. Nicholas Carleton

**Affiliations:** Anxiety and Illness Behaviours Laboratory, University of Regina, Regina, SK, Canada

**Keywords:** public safety personnel, stigma, mental health knowledge, resilience, help-seeking

## Abstract

**Introduction:**

Public safety personnel (PSP; e.g., communications officials [e.g., 911 call center operators/dispatchers], correctional service employees, firefighters, paramedics, police officers) experience an elevated risk for mental disorders due to inherent work-related stress. Several programs have been designed to increase mental health knowledge, intending to reduce stigma, and increase mental health service help-seeking (e.g., resilience training); however, extant programs have not demonstrated sustained improvements for PSP mental health. The current study assessed levels of mental health knowledge, stigma, and service use intentions in a sample of Canadian PSP and compared trends to published estimates of mental health symptoms across PSP categories to inform future programming.

**Methods:**

PSP completed questionnaires assessing mental health knowledge, stigma against coworkers with mental illness, and professional service use intentions. Correlations among variables and one-way analyses of variance were conducted to assess differences among categories. PSP were categorized into six categories for comparison: communication officials, correctional workers, firefighters, municipal/provincial police, paramedics, and Royal Canadian Mounted Police (RCMP).

**Results:**

There were significant differences between categories for each variable. Correctional workers reported the most mental health knowledge, least stigma, and highest intentions to use mental health services, and the highest positive screens for mental disorders. Conversely, firefighters reported the lowest mental health knowledge, highest stigma, and lowest willingness to seek professional help, and the lowest prevalence of positive screens for mental disorders.

**Discussion:**

The results contrast previously hypothesized associations among mental health variables where education, stigma reduction, and help-seeking have been expected to improve mental health. The discrepant results offer potentially critical information for organizational policies to better support PSP. Individuals reporting mental health symptoms may be a more appropriate target audience for intervention strategies, given the possible, crucial role personal experience plays in increasing mental health knowledge, and ultimately, encouraging help-seeking.

## Introduction

Canadian public safety relies on services from dedicated professionals known collectively as public safety personnel (PSP; e.g., communications officials [e.g., 911 call center operators/dispatchers], correctional service employees, firefighters, paramedics, police officers; Canadian Institute for Public Safety Research and Treatment [CIPSRT], 2019; Oliphant, 2016). Repeated exposures to potentially psychologically traumatic events (PPTEs) increase the risk of PSP developing symptoms of mental disorders (Carleton et al., 2019). Recent estimates suggest that almost 45% of PSP screened positive for one or more mental disorders^1^ (Carleton et al., 2018a), whereas diagnostic rates for the general population are closer to 10% (Statistics Canada, 2012). The differences evidence the increased vulnerability for PSP potential and help to quantify the magnitude of their mental health challenges.

Exposure to PPTEs may be an unavoidable vocational reality for PSP. Consequently, increased efforts are being dedicated to securing improved mental health education, prevention, and treatment for PSP (e.g., Authors, 2016; Carleton et al., 2020b). The ultimate goal of implementing such mental health programming is to reduce the prevalence of mental disorders by increasing knowledge, reducing stigma, and therein promoting early interventions. Indeed, contemporary resilience training programs are designed to increase help-seeking behaviors (Papazoglou and Andersen, 2014). Positive benefits with small to moderate effects have been identified in longitudinal (Carleton et al., 2018c) and cross-sectional (Leppin et al., 2014; Robertson et al., 2015; Carleton et al., 2020b) studies of resilience training programs.

Published program evaluation results with data from PSP have been relatively limited. For example, the Road to Mental Readiness (R2MR) program was adapted from the Canadian Armed Forces for Canadian police to reduce mental health stigma and improve mental health literacy (Carleton et al., 2018c). Police participating in the R2MR program did not report statistically significant changes in mental health knowledge, resilience, or symptoms compared to pre-intervention (Carleton et al., 2018c). Small, but statistically significant, reductions in stigma were reported immediately following the training, but were not sustained at the 6- or 12-month follow-ups (Carleton et al., 2018c). Nonetheless, participants reported that the R2MR program was helpful for improving communication and facilitated more open dialogs about mental health 1 year post-training through open-ended comments (Carleton et al., 2018c). Effectiveness evaluations of R2MR with several PSP categories (i.e., corrections workers, emergency services, fire services, paramedics, and police) have also evidenced statistically significant improvements in resilience skills and intentions toward seeking help, as well as reductions in stigma up to 3 months post-training (Szeto et al., 2019); however, R2MR training outcomes have varied by PSP category, gender, and supervisors/frontline staff (e.g., relatively more improvement for police in stigma; Szeto et al., 2019). A randomized controlled trial testing the effectiveness of R2MR with 65 military platoons highlighted the influence of the larger organizational climate and concluded that beneficial effects of R2MR require high fidelity program delivery (Fikretoglu et al., 2019b). That said, the available cross-sectional evidence suggests that participation in any of several mental health training programs (i.e., critical incident stress management/debriefing [CISM/CISD], mental health first aid, peer support, R2MR) can be associated with more willingness to access support and lower likelihood of screening positive for a mental disorder (Carleton et al., 2019).

Mental health literacy reflects knowledge about mental disorders (Jorm, 2012). Highly literate individuals may know better how to manage mental disorders, recognize the development of disorders, and identify help-seeking or treatment options (Reavley and Jorm, 2011; Jorm, 2012). Individuals may acquire mental health knowledge from personal experiences. For example, a sample of soldiers who self-reported mental health problems demonstrated higher mental health literacy than soldiers without mental health problems (Thomas et al., 2016). Similarly, individuals who can relate to the mental health information presented as part of educational programming may find it more salient and then report higher mental health literacy. PSP must have adequate knowledge about mental disorders in order to recognize indicators of distress that may require more support and then what treatment options are effective and available; however, the lack of sustained changes to PSP mental health knowledge following R2MR training suggests that a one-time presentation of mental health information may be insufficient for sustained improvements in mental health service use acceptance.

Stigma may be a distinct barrier to treatment-seeking behavior. Negative attitudes toward mental disorders can hinder recognition and help-seeking behaviors (Clement et al., 2015; Cheng et al., 2018). Self-stigma and public stigma have both been inversely associated with help-seeking (Corrigan, 2004; Conner et al., 2010). Having a mental disorder is often associated with being perceived as incapable, incompetent, weak, and a failure (Violanti, 2010; Caputo and Rouner, 2011). Pressure to maintain a strong persona is often greater among PSP organizations (Corsianos, 2011), which may lead PSP to avoid accessing professional services for fear of retribution by peers or administration (Blum, 2000; Karaffa and Koch, 2015; White et al., 2016; Wheeler et al., 2018). PSP who are mistrustful of peers with mental disorders (e.g., perceiving peers as unstable or a risk to personal safety on calls) will not feel comfortable disclosing mental health struggles, further reducing social support and treatment-seeking behavior (Violanti, 2010; Wilmoth, 2014). Mental health stigma may also influence mental health symptom reporting, hindering realistic prevalence estimates, limiting overall resource availability (Henderson et al., 2016), and creating a cycle that further impedes treatment-seeking behavior.

The ultimate goal of increasing mental health knowledge and reducing associated stigma is to increase PSP willingness to seek treatment when needed. Earlier intervention and treatment-seeking behaviors might reduce the burden of mental disorders. Despite reporting having access to professional mental health supports, most PSP reported only intending to access such support as a last resort (Carleton et al., 2019). Presenting mental health education to increase mental health literacy has not yet produced sufficiently robust effects to protect PSP from experiencing mental disorder symptoms (Carleton et al., 2018c). Incremental reductions in stigma are possible and noteworthy (Carleton et al., 2018c); however, a connection between improvements in attitudes regarding mental disorders and action (i.e., treatment-seeking behavior) has yet to be identified. The available research has demonstrated help-seeking attitudes as better predicted by mental health knowledge than stigma (Cheng et al., 2018), which suggests that additional research is warranted and may lead to better support systems for PSP mental health.

A dearth of research assessing resilience programs has led to insufficient knowledge for informing best practices for supporting PSP mental health. If resilience programs increase mental health knowledge, reduce stigma, and increase willingness to seek help when needed, mental health symptoms should be reduced. Researchers, clinicians, and PSP leaders require more information to identify appropriate targets for intervention.

### Current Study

The current study was designed to determine the relationship among mental health knowledge, stigma against peers in the workplace, and service use intentions in a nationally representative sample of PSP. In addition, differences among PSP categories were examined for evidence of systemic or personal differences across occupations. Hypotheses were as follows: (1) as mental health knowledge increases and stigma decreases, service use intentions will increase for the total sample and occupational categories; and (2) trends in mental health knowledge, stigma, and service use intentions will correspond to the mental health prevalence estimates for each category (Carleton et al., 2018a). Accordingly, firefighters were expected to demonstrate the highest mental health knowledge, lowest stigma, and highest willingness to seek mental health services because they have reported the lowest prevalence of positive screens for mental disorders (Carleton et al., 2018a). In contrast, correctional workers had the highest proportion screening positive for at least one mental disorder (Carleton et al., 2018a), which suggested that they would report the lowest mental health knowledge, highest stigma, and lowest service use intentions.

## Materials and Methods

### Participants and Procedure

Participants were recruited as part of a pan-Canadian online study of PSP available in English and French from September 2016 to January 2017. The survey was collaboratively designed by researchers from the University of Regina and the Public Safety Steering Committee (PSSC) of the Canadian Institute for Public Safety Research and Treatment (CIPSRT). The survey link was distributed through email to currently employed PSP by the PSSC and provincial and municipal PSP agencies. Participation was voluntary and anonymous, and the study was approved by the University of Regina Institutional Research Ethics Board (File #2016-107). Details of the study procedure have been published elsewhere (Carleton et al., 2018a, 2019; Ricciardelli et al., 2018a). In total, *n* = 8,520 began the survey and *n* = 4,108 (48.2%) completed all of the survey questions associated with the current analyses. PSP participants were assigned to one of six categories for analyses: communication officials (e.g., 911 call center operators/dispatchers), correctional workers, federal police (i.e., Royal Canadian Mounted Police: RCMP), firefighters, municipal/provincial police, and paramedics.

### Measures

#### Mental Health Knowledge Scale

The Mental Health Knowledge Scale (MAKS; Evans-Lacko et al., 2010) is a 15-item self-report questionnaire. The first six items assess beliefs about mental health (e.g., “People with severe mental health problems can fully recover”) proceeded by nine items designed to assess participants’ level of recognition and familiarity with various mental health conditions (e.g., stress; posttraumatic stress disorder). All items were rated on a 5-point Likert scale ranging from 1 (*strongly disagree*) to 5 (*strongly agree*). Data support the test–retest reliability (Evans-Lacko et al., 2010) and the Cronbach’s *α* was *α* = 0.71 for the current sample.

#### Open Minds Survey for Workplace Attitudes

The Open Minds Survey for Workplace Attitudes (OMS-WA; Szeto et al., 2013) is a self-report questionnaire that includes 11 items designed to measure attitudes regarding avoidance and danger/unpredictability toward people with mental illness. Items such as “I would try to avoid a coworker with a mental illness” were rated on a 5-point Likert scale ranging from 1 (*strongly disagree*) to 5 (*strongly agree*). Higher scores indicate higher mental health stigma in the workplace. The Mental Health Commission of Canada employs the OMS-WA as a standard metric for stigma. The OMS-WA had a Cronbach’s *α* = 0.90 in the total sample.

#### Mental Health Service Use Questionnaire

Mental Health Service Use Questionnaire (MHSUQ) is a 4-item self-report questionnaire designed to measure willingness to seek professional help for mental illness. Items (e.g., “If I developed mental health problems, I would want to seek mental health treatment from a professional”) are rated on a 7-point Likert scale, ranging from 1 (*strongly disagree*) to 7 (*strongly agree*). The MHSUQ is derived from the 76-item CAF-R-MHSUQ (Fikretoglu et al., 2019a) and consistent with questions regularly used in Statistics Canada surveys to assess mental health service use. The Cronbach’s *α* for the MHSUQ was *α* = 0.95 in the current sample.

### Analyses

First, zero-order correlations were conducted among mental health knowledge, stigma, and service use with the entire sample and then within each PSP category to assess the variable interrelationships. Second, multivariate analysis of covariance (MANCOVA) was conducted to determine whether there were significant differences in mean mental health knowledge, stigma, or service use intention across PSP categories while accounting for age. Statistical significance was set at *p* ≤ 0.05. If there was a statistically significant difference, pairwise comparisons with a Bonferroni correction were used to determine which categories were significantly different. All analyses were conducted using SPSS version 26 software (IBM Corp., Armonk, NY, United States).

## Results

### Demographics and Correlations

The demographic variables for each PSP category and the total sample are presented in Table 1. The survey allowed for sex (binary) and gender (including non-binary) responses, although no respondents identified as non-binary. No statistically significant differences were observed between sex and gender; as such, only the sex variable was retained in the analyses. As no patterned differences emerged between career and volunteer firefighters, they remained grouped together. For the total sample, mental health knowledge and service use intentions correlations were statistically significant, but the relationship was relatively small (*r* = 0.191, *p* < 0.001). Stigma was inversely statistically significantly correlated with knowledge (*r* = −0.323, *p* < 0.001) and service use attitudes (*r* = −0.176, *p* < 0.001). The correlations were in the same direction and statistically significant within each PSP category (*p’*s < 0.04). Skewness and kurtosis were effectively normal for all measures. The results suggest that higher levels of mental health knowledge were associated with lower stigma and higher willingness to seek professional mental health services.

**TABLE 1:** Demographics by PSP category.

		**Mean/% (*n*)**					
	**Total *n* = 4180**	**Municipal/provincial police *n* = 1065**	**RCMP *n* = 1070**	**Firefighters *n* = 641**	**Paramedics *n* = 603**	**Corrections *n* = 592**	**Comm. officials *n* = 209**
Age, years	43.35	43.28	43.35	46.11	40.28	44.32	41.26
**Sex**							
Male	67.8 (2826)	72.7 (772)	73.8 (786)	92.8 (595)	60.7 (366)	44.2 (260)	22.5 (47)
Female	32.2 (1340)	27.3 (290)	26.2 (279)	6.9 (44)	39.3 (237)	55.8 (328)	77.5 (162)
**Location (province)**							
Western Canada	54.3 (2232)	43.9 (467)	63.7 (648)	53.5 (342)	62.3 (375)	52.9 (308)	44.2 (92)
Eastern Canada	33.8 (1392)	47.2 (502)	15.6 (159)	41.9 (268)	22.9 (138)	40.2 (234)	43.8 (91)
Atlantic Canada	11.9 (489)	8.9 (95)	20.7 (211)	4.5 (29)	14.8 (89)	6.9 (40)	12.0 (25)

*PSP = public safety personnel; RCMP = Royal Canadian Mounted Police; Corrections = correctional workers; Comm. officials = communication officials.*

### Differences in Knowledge, Stigma, and Service Use Across PSP Categories

The MANCOVA indicated statistically significant differences between the PSP categories in combined mental health knowledge, stigma, and service use, after controlling for age, *F* (15, 11453) = 12.24, *p* < 0.001, Wilks’ Λ = 0.957. For mental health knowledge, differences were statistically significant across PSP categories, *F* (5, 4174) = 17.54, *p* < 0.001, ω = 0.16. For stigma, differences were statistically significant across PSP categories, *F* (5, 4174) = 24.26, *p* < 0.001, ω = 0.14. For service use, differences were statistically significant across PSP groups, *F* (5, 4174) = 5.52, *p* < 0.001, ω = 0.07. Several statistically significant differences were identified across PSP categories for each dependent variable, as indicated by superscripts in Table 2. No statistically significant interactions between sex and PSP categories were found; as such, we did not control for sex in the current analyses.

**TABLE 2:** Means for MAKS, OMS-WA, and MHSUQ.

	***M* (*SD*)**						
	**Total sample**	**Municipal/provincial police**	**RCMP**	**Firefighters**	**Paramedics**	**Correctional workers/officers**	**Communication officials**
MAKS	47.06 (4.35)	46.70(4.22)b,c	47.20(4.33)b,c	45.97(4.26)d	47.53(4.47)a,b	48.01(4.45)a	47.48(3.81)a,b,c
OMS-WA	21.13 (7.31)	21.94(7.46)b	20.62(7.12)a	23.36(7.31)c	19.97(6.90)a	19.82(7.25)a	19.88(6.87)a
MHSUQ	23.30 (5.91)	23.59(5.79)a,b	22.88(6.21)b	23.20(5.92)a,b	22.68(6.12)b	23.93(5.47)a	24.29(5.21)a

*MAKS = Mental Health Knowledge Schedule; OMS-WA = Open Minds Survey for Workplace Attitudes; MHSUQ = Mental Health Service Use Questionnaire; RCMP = Royal Canadian Mounted Police. Letters in the cells indicate categories of public safety officers that are significantly different from one another at *p* < 0.05; e.g., correctional workers “a” reported statistically significantly higher mental health knowledge than municipal/provincial police “b, c,” and RCMP “b, c,” but were not statistically significantly different compared to communication officials “a.”*

## Discussion

The present study examined the relationship among mental health knowledge, stigma against peers in the workplace, and service use intentions in a nationally representative sample of PSP. Resilience training is commonly used within PSP agencies to minimize barriers to seeking treatment, primarily through reducing stigma (Papazoglou and Andersen, 2014). Theoretically, as mental health knowledge increases, stigma should be reduced, and intentions to access mental health services if needed should be increased. Results from the current cross-sectional analysis generally supported the hypothesized relationship patterns between mental health knowledge, stigma, and intended service use.

The relationships between knowledge, stigma, and service use intentions were generally consistent with our hypotheses across PSP categories (i.e., communications officials, correctional workers, firefighters, municipal/provincial police). Correlational strengths among the variables across PSP categories differed, suggesting potentially important nuances for PSP based on occupation. For example, paramedics reported not only high mental health knowledge and low stigma but also the lowest intentions to seek help if needed compared to other PSP categories. Paramedics reporting high mental health knowledge might intuitively suggest that paramedics will also have a high willingness to engage in help-seeking; however, the current evidence suggests that other variables may be influencing help-seeking decisions. Paramedics may have reported lower service use intentions because of self-stigma, personal experience, or systemic barriers. If paramedics recognize mental disorders and effective treatment options, but still choose not to seek the necessary personal help, the cause may be self-stigmatization (Corrigan, 2004). The study assessed stigma toward others (in which paramedics scored low), but paramedics may be less empathetic toward their own struggles (Jones, 2017). Paramedics are frequently called for mental health emergencies and have likely acquired high mental health literacy (Elliott, 2013; Holmes, 2019). Professional experiences that increase mental health literacy may be cataloged as job-specific; therefore, for a myriad of different possible reasons, paramedics may not be applying mental health knowledge acquired on calls to their own personal experiences to encourage help-seeking. For example, repeated experiences with mental health calls may reduce paramedics’ confidence in the beneficial value of mental health services (Roberts and Henderson, 2009) or underscore the limitations and barriers to accessing evidence-based mental health care and clinicians with experience working with PSP. Additional research appears warranted to facilitate help-seeking among paramedics.

Correctional workers have previously been evidenced as reporting the highest overall prevalence of positive screens for mental disorders (Carleton et al., 2018a; Ricciardelli et al., in press). The current results demonstrated that correctional workers also reported the highest mental health knowledge, lowest stigma, and highest intentions to seek mental health services. The high prevalence of positive screens is inconsistent with notions that increasing knowledge may protect mental health (Jorm, 2012). Correctional workers are responsible for people held in custody, may be expected to provide mental health support (Dvoskin and Spiers, 2004), and frequently experience workplace PPTE (Carleton et al., 2019), including violence (Ricciardelli et al., 2018b). To cope, correctional workers consider the heightened violence as “part of the job” (Ricciardelli et al., 2018b). Still, correctional workers reported the highest prevalence of suicidal behaviors compared to all categories except paramedics (Carleton et al., 2018b) and die by suicide at a rate 39% higher than the general population (Kochanek et al., 2015). Correctional workers report having the most mental health knowledge and still have the largest proportions screening positively for one or more mental disorders; as such, a different protective mechanism may be at work than current resilience training ideology proposes (e.g., Papazoglou and Andersen, 2014). Rather than learning from psychoeducation, PSP may be gaining mental health knowledge through personal experience. Lower stigma may also encourage an environment wherein individuals are more willing to discuss their mental health concerns, meaning more people may be aware of their colleagues or their own struggles. Increased awareness may also increase an individual’s ability to identify and then self-report on their symptoms. The results among firefighters were inconsistent with hypotheses. Specifically, firefighters reported the lowest mental health knowledge and the highest stigma compared to other PSP categories, despite reporting the lowest prevalence of positive screens for mental disorders (Carleton et al., 2018a). The unexpected results suggest that other factors deserve attention (e.g., the impact of peer support, regular exercise during shiftwork). Overall, PSP struggling with symptoms of mental illness may be the most appropriate target audience for psychoeducation intended to reduce mental health stigma, encourage service use, and promote wellbeing.

The PSP groups reporting the highest mental health knowledge were predominantly comprised of females and had previously been identified as having worse mental health outcomes (Carleton et al., 2018a); specifically, the correctional worker and communication official categories were mostly female. Despite the lack of statistically significant effects between sex and PSP categories, differences associated with the sex distributions may provide valuable information about public safety mental health. Female PSP may be exposed to more sexualization, disrespect, sexually charged threats, and violence than their male counterparts (Pogrebin and Poole, 1997; Batton and Wright, 2019). Females are also more likely to be aware of their emotional states and more able to report on their symptoms on a self-report questionnaire (Mankus et al., 2016); therefore, having increased mental health knowledge may increase the likelihood that a person can and will report difficulties. Pressures may also exist at work for female PSP to act stoically on the job, which may compromise their coping or their overall resilience (Morash et al., 2006; Batton and Wright, 2019). Definitive conclusions underlying sex differences may require a series of qualitative studies to inform subsequent quantitative studies specifically designed to assess how gender influences mental health in and across PSP categories.

### Strengths and Limitations

The current study has several strengths and limitations that provide directions for future research. First, a large, representative sample of Canadian PSP was identified in the current study and allowed for comparisons across public safety occupations rather than focused attention to one category. Second, the study investigated three variables that have not yet been studied in combination to assess theoretical explanations for variability in resilience. Future investigations of such theoretical models should use longitudinal data to assess for differences across PSP categories. Third, the survey was not deployed using a stratified sample, limiting the generalizability of the results. Future studies should use systemic recruitment to minimize self-selection biases and ensure a random representative sample. Fourth, the use of correlational, cross-sectional self-report data limits the ability to determine causality regarding differences. Longitudinal research designs with clinician-administered diagnostic assessments appear warranted. Fifth, several of the self-report measures do not have evidence for cutoff scores regarding clinically significant levels of mental health knowledge or stigma; as such, there is no way to assess the real-life impact of the self-reported attitudes on behaviors. Actual behavioral metrics, possibly identified through medical record reviews, may provide important corollary information.

Further research is required to determine the best practices for supporting help-seeking among PSP. Contemporary attempts to use education for increasing mental health knowledge, decreasing stigma, increasing resilience, and improving mental health appear to have limited impact (Carleton et al., 2018c), possibly due to important differences in occupational stressors or PPTE exposure types (Carleton et al., 2019, 2020a). The nature of PSP work may make prevention of mental health injuries unrealistic, which underscores the importance of having pervasive proactive and reactive strategies that are readily engaged and effective. Further research on mental health training programs may help to advance the effectiveness of training and therein help-seeking and overall mental health. Similarly, researchers may design longitudinal studies to assess whether ongoing training is requisite for lasting effects (Carleton et al., 2018a). In addition, there may be opportunities for improvements at the systemic and organizational levels to reduce stigma (e.g., regular wellness checks, peer-support programs, family programming) and other barriers to care (e.g., prohibitive costs for services, facilitating earlier interventions), which may improve PSP mental health (Carleton et al., 2020b). Such improvements would still benefit from increased understanding about the current state of knowledge, stigma, and service use intentions among PSP. The current study offers valuable, immediate implications for policy and procedures; for example, psychoeducation programs intended to reduce mental health stigma, encourage service use, and promote wellbeing may be particularly beneficial for PSP who are currently struggling with symptoms of mental health challenges.

## Data Availability Statement

The datasets generated for this study will not be made publicly available as data security procedures were made explicit by all parties prior to collection. Output from analyses with de-identified data may be available.

## Ethics Statement

The studies involving human participants were reviewed and approved by the University of Regina Institutional Research Ethics Board (file No. 2016-107). The patients/participants provided their written informed consent to participate in this study.

## Author Contributions

RC and RK: conceptualization. RC, RK, and AS: methodology, analysis interpretation, and writing – review and editing. RK and AS: data analysis and writing–original draft preparation. RC: supervision. RK: project administration. All authors approved the submitted version of the manuscript.

## Conflict of Interest

The authors declare that the research was conducted in the absence of any commercial or financial relationships that could be construed as a potential conflict of interest.

## Footnotes

1 All screening measures were validated for identification of individuals who may require further clinical attention and are not definitive diagnostic tools.
